# Pseurotin D Induces Apoptosis through Targeting Redox Sensitive Pathways in Human Lymphoid Leukemia Cells

**DOI:** 10.3390/antiox10101576

**Published:** 2021-10-05

**Authors:** Eva Mosejová, Rebeka Bosnjakovic, Lukáš Kubala, Ondřej Vašíček

**Affiliations:** 1 Institute of Biophysics of the Czech Academy of Sciences, 612 65 Brno, Czech Republic; mosejova@ibp.cz (E.M.); rebekabosnjakovic94@gmail.com (R.B.); kubalal@ibp.cz (L.K.); 2 Institute of Experimental Biology, Faculty of Science, Masaryk University, 625 00 Brno, Czech Republic; 3 International Clinical Research Center, St. Anne’s University Hospital, 656 91 Brno, Czech Republic

**Keywords:** pseurotin D, proliferation, apoptosis, mitochondrial activity, reactive oxygen species, lymphoma

## Abstract

Chronic lymphocytic leukemia (CLL) is the most prevalent lymphoid malignancy in many geographical regions of the world. Pseurotin D, a secondary metabolite of fungi, represents a group of bioactive natural products with a newly ascribed range of interesting biological activities. The purpose of this study was to bring new insights into the mechanism behind the effects of pseurotin D on MEC-1 cells as a representative CLL cell line, with a particular focus on selected signaling pathways important in the proliferation of cells and targeting mitochondrial metabolism. Our results showed that pseurotin D was able to significantly inhibit the proliferation of MEC-1 cells and arrested them in the G2/M cell cycle phase. In addition, pseurotin D was able to induce apoptosis. We found that all of these effects were associated with a change in mitochondrial membrane potential and the production of mitochondrial reactive oxygen species (ROS). We showed for the first time that pseurotin D suppresses MEC-1 cell proliferation and induces apoptotic cell death via induction of the collapse of the mitochondria respiratory chain and the ROS-related caspase pathway. Our results show the pseurotins family as promising compounds which could serve as a basis for the development of new compounds in the treatment of lymphoma.

## 1. Introduction

Chronic lymphocytic leukemia (CLL) is the most common lymphoid malignancy in many geographical areas of the world. The estimated annual incidence of CLL in the European population is 4.9/100,000/year. CLL treatment is improving rapidly, but is still not curative. The size of the CLL patient population in the near future is likely to increase due to an aging population together with the chronic nature of the disease [1]. The current treatment consists mainly of a combination of anticancer chemotherapy and radiotherapy focusing on the inhibition of BCR signalization and other important pathways, or the induction of apoptosis [2,3,4].

Most recently, due to elevated mitochondrial metabolism in CLL cells, mitochondria have also become promising targets for cancer therapy. Increased oxidative phosphorylation comes with a higher production of ROS, which is compensated by better antioxidative mechanisms. However, exceeding this antioxidative capacity by even a small amount can promote intolerable oxidative stress and apoptosis [5]. Compounds, such as 2-methoxyestradiol, cisplatin or buthionine sulfonimine which promote ROS production or disrupt redox homeostasis may induce the death of cancer cells and inhibit tumor growth. [6]. Therefore, newly discovered compounds target electron transport chain or mitochondrial metabolism [7].

In addition to typical cytostatics preventing cell division, new drugs are being introduced that affect the Janus kinase (JAK)/signal transducer and activator of transcription (STAT) and mitogen-activated protein kinase (MAPK) signaling pathways, which are important in the regulation of cell proliferation [8] and the targeting of mitochondria metabolism [7]. Blocking the JAK/STAT signaling pathway in cancer cells can suppress the expression of target genes that control essential cell functions and hinder cancer cells from evading growth control mechanisms, such as apoptosis and invasion [9]. Targeting STAT3 activation inhibits tumor growth and metastasis both in vitro and in vivo without affecting normal cells [10].

Together with the JAK/STAT signaling pathway, the MAPK pathway also plays an important role in cell proliferation, differentiation, apoptosis, angiogenesis, and tumor metastasis. Hyperactivation of kinases ERK1 and ERK2 plays a major role in cancer development, progression, and the survival of cancer cells, with the Ras/Raf/MAPK (MEK)/ERK pathway being recognized as the most important one [11]. JAK/STAT signaling may act either directly or indirectly by triggering the activation of nuclear factor-κB (NF-κB) [12]. The functional importance of mitochondrial STAT3 has been extensively explored. STAT3 has been linked to the control of the electron transport chain and production of ATP, the regulation of mitochondrial encoded RNA, the modulation of reactive oxygen species (ROS) generation, Ras transformation, and cellular growth [13,14,15].

Small organic molecules bring new possibilities in studying the modulation of important signaling pathways and also have a potential for the modulation of specific physiological processes in cell biology. Secondary metabolites from nature may be a source for the development of new bioactive small molecules used as drugs in human medicine [16,17].

Pseurotins, the secondary metabolites of fungi, have important biological activities. In addition to their antifungal and antibiotic activities [18], pseurotins were shown to modulate cell differentiation [19], to possess anti-angiogenic activity and to inhibit endothelial cell migration [20,21,22], and to regulate cellular metabolism enzymes [23]. Interestingly, significant effects of pseurotins related to modulation of the JAK/STAT and MAPK signaling pathways were observed in immune cells; when the natural pseurotins A and D significantly inhibited the proliferation of murine macrophages, this accompanied by lower level of cyclins and downregulation of mitochondrial respiration [24]. Further, these natural pseurotins and a collection of fully synthetic pseurotin analogs were shown to inhibit the production of immunoglobulin E and the proliferation of mouse B-cells via inhibition of the phosphorylation of STAT proteins [17]. Additionally, our previously published data showed that pseurotin D inhibits the activation and proliferation of human T-cells [25].

In light of these facts, the purpose of this study was to investigate the potential effects of pseurotin D on the proliferation, mitochondrial activity, and redox potential balance of MEC-1 cell line (representative human chronic leukemia B cells) and to bring new insights into the usefulness of this compound for the development of new compounds in the treatment of lymphoma.

## 2. Materials and Methods

### 2.1. Materials

Pseurotin D (cat # BVT-0426) was purchased from Adipogen Life Science (San Diego, CA, USA). A stock solution of pseurotin was prepared in dimethyl sulfoxide (DMSO) at 10 mM concentration. Final concentrations 1–75 µM of pseurotin D were diluted in culture medium. DMSO at final dilutions was not toxic to MEC-1 cells, as proven by 3-(4,5-dimethylthiazol-2-yl)-2,5-diphenyltetrazolium bromide (MTT) tests (Figure S1).

### 2.2. Cell Culture

MEC-1 cells (American Type Culture Collection, VA, USA), a stable cell line of chronic lymphocytic leukemia, were grown in Iscove’s Modified Dulbecco’s Medium (IMDM; Gibco, Waltham, MA, USA) with 10% fetal bovine serum (FBS; Gibco, Waltham, MA, USA), penicillin and streptomycin (both 100 μg/mL, Sigma, St. Louis, MO, USA) at 37 °C in 5% CO_2_.

### 2.3. Cell Viability and Cytotoxicity

Total number of cells and the amount of metabolically active cells were the basis for the evaluation of cell viability. The number of cells was counted on a CASY hemocytometer (OLS OMNI Life Science GmbH & Co KG, Bremen, Germany) [25]. The amount of metabolically active cells was determined by analysis of the total amount of reduced MTT to its insoluble formazan in cell lysates, which reflects the number of metabolically active viable cells in culture, as described in Moosova et al. [26]. Cells were incubated in a 24-well plate at a concentration of 2.5 × 10^5^ cells per well and incubated with pseurotin D (concentration range 1–75 µM) for 24, 48, 72, and 96 h at 37 °C and under a 5% CO_2_ atmosphere. At the end of the incubation period, culture medium with cells was collected from the wells; 20 µL of cell suspension was used for cell counting, and the rest of the medium was centrifuged, the pellets used for MTT assay and the supernatant for lactate dehydrogenase (LDH) assay.

A stock solution of MTT (2.5 mg/mL) was diluted in culture media to a final concentration of 0.25 mg/mL. Cells were resuspended in cell culture medium supplemented with MTT and incubated for another 3 h. The medium was removed and formazan from cells was extracted with 100 µL of 10% Triton X-100 in 0.1 M HCl per well on a shaker for 15 min. The extract was clarified by short spin centrifugation and the absorbance was read at 570 nm on a SPECTRA Sunrise microplate reader (Tecan, Mannedorf, Switzerland).

Possible cytotoxic effects of pseurotin were determined by measuring LDH activity released into cell media. For detection of LDH activity the Cytotoxicity Detection KitPLUS (Roche, Pleasanton, CA, USA) was used, as described previously [27]. Briefly, 100 μL of supernatant was mixed with the 100 μL of reaction mixture and incubated at room temperature (RT). The absorbance was measured by SPECTRA Sunrise microplate reader (Tecan, Mannedorf, Switzerland) at 490 nm. MEC-1 cells lysed by adding 15 μL of lysis buffer were used as a positive control.

### 2.4. Apoptosis of Cells

The apoptosis of MEC-1 cells was determined by means of Annexin V Apoptosis Kit-FITC (Exbio, Prague, Czech Republic); FITC active caspase-3 Apoptosis kit (BD Bioscience, Carlsbad, CA, USA); and the detection of PARP, cleaved caspase-3 and -9, and the fragmentation of nuclei. To analyze apoptosis over a period of 96 h, cells were incubated in a 24-well plate at a concentration of 2.5 × 10^5^ cells per well and incubated with pseurotin D (at concentrations of 10, 15, 25, and 50 µM) for 24, 48, 72, and 96 h. After exposure, the reaction mixture of Annexin V (1:150 Annexin V-FITC and 1× Annexin V binding buffer) was added and incubated for 20 min in the dark. Immediately before analysis by flow cytometry (FACSVerse™, BD Bioscience, Carlsbad, CA, USA), 2 μL of propidium iodide (PI, final concentration 1 mg/mL) was added to each sample.

To analyze apoptotic cells in individual phases of the cell cycle, cells were incubated in a 24-well plate at a concentration of 2.5 × 10^5^ cells per well and incubated with pseurotin D (at concentrations of 10, 15, 25, and 50 µM) for 24 h. After exposure, the cells were fixed and permeabilized for 20 min with the Cytofix/Cytoperm solution included in the kit. After permeabilization, the cells were stained for 30 min with FITC active caspase-3 antibody and for another 30 min with PI solution (1M Tris pH 8, 0.7 mg/mL RNase, 1% Triton X-100, 50 µg/mL PI). Finally, the stained cells were measured by a flow cytometer (FACSVerse™, BD Bioscience, Carlsbad, CA, USA).

To analyze the proteins PARP, BAX, and cleaved caspase-3 and -9, cells were incubated in a 24-well plate at a concentration of 2.5 × 10^5^ cells per well and incubated with pseurotin D (at concentrations of 10, 15, 25, and 50 µM) for 24 h. After the incubation period, the culture medium was removed and cells were stored in a freezer for further processing (see Western blot paragraph).

To detect the fragmentation of DNA, cells were incubated in a 24-well plate at a concentration of 2.5 × 10^5^ cells per well and incubated with pseurotin D (at concentrations of 25 and 50 µM) for 24 and 48 h. After the incubation period, the cells were stained using the protocol described previously [28]. Briefly, the cells were washed inphosphate-buffered saline (PBS), centrifuged (300 g/5 min/RT), transferred into an Eppendorf tube, and fixed in 4% formaldehyde for 20 min at RT. They were then permeabilized with 0.1% TritonX-100 for 30 min at RT and subsequently washed in 1× PBS. Nuclei were counterstained with DAPI (1 μg/mL) and transferred onto microscopic plates (MakTek Corporation, Ashland, MA, USA) coated with poly-L-lysine (sc-286689, Santa Cruz Biotechnology, Santa Cruz, CA, USA). Images were acquired using a confocal microscope (TCS SP5; Leica, Wetzlar, Germany) equipped with a 63× 1.4 oil immersion objective.

### 2.5. Cell Cycle Analysis

The cell cycle of MEC-1 cells was determined by using the Click-iT EdU flow cytometry assay kit (Life Technologies, Carlsbad, CA, USA) [29] and by the detection of cyclins [24]. The cells were synchronized for 24 h in IMDM medium without FBS. Synchronized cells were incubated in a 24-well plate with pseurotin D (at concentrations of 10, 15, and 25 µM) in complete medium for 24 h. Two hours before the end of exposure, 5 μL of 1 mM EdU were added. After the incubation period, cells were fixed, permeabilized, stained by a reaction mixture of fluorescent dye, and measured by a flow cytometer (FACSVerse™, BD Bioscience, Carlsbad, CA, USA).

To analyze the protein levels of cyclin A and B1, cells were synchronized in IMDM medium without FBS for 24 h. Synchronized cells were incubated in a 24-well plate at a concentration of 5 × 10^5^ cells per well and incubated with pseurotin D (at concentrations of 10, 15, 25, and 50 µM) for 24 h. After the incubation period, the culture medium was removed and the cells were frozen for further processing (described in Western blot analysis paragraph).

### 2.6. Western Blot Analysis

To analyze the protein phosphorylation of members of the selected signaling pathways, cells were incubated in Eppendorf tubes at a concentration of 1 × 10^6^ cells per tube with pseurotin D (at concentrations of 10, 15 and 25 µM). Pseurotin D was added for 6 h. At the end of the incubation period, the culture medium was removed and cells were stored in a freezer for further processing.

After the treatment procedure, MEC-1 cells were lysed using a lysis buffer containing phenylmethylsulfonyl fluoride. The protein concentration was determined using BCA™ protein assay (Pierce, Waltham, MA, USA) with BSA used as a standard. The expressions of target proteins were quantified by western blot analysis, as described previously [30]. The protein (22.5 µg) from each lysate supplemented with Laemmli buffer was subjected to SDS-polyacrylamide gel electrophoresis, using 10% running gel for the detection of cyclins and 12.5% for the detection of cleaved caspases, PARP, and BAX. The separated proteins were transferred to PVDF membranes that were blocked in 5% non-fat milk in Tris-buffered saline and 0.1% Tween 20 (TBS-T). Then, the membranes were incubated with monoclonal antibodies against vinculin (1:10,000, #13901S, Cell signaling technologies, Danvers, MA, USA), STAT3 (1:300; #4904, Cell signaling technologies, Danvers, MA, USA), p-STAT3 (1:300; #9145, Cell signaling technologies, Danvers, MA, USA), ERK1/2 (1:1000; #4695S, Cell signaling technologies, Danvers, MA, USA), p-ERK1/2 (1:1000; #9101S, Cell signaling technologies, Danvers, MA, USA), p38 (1:1000; #9212S, Cell signaling technologies, Danvers, MA, USA), p-p38 (1:1000; #4511S, Cell signaling technologies, Danvers, MA, USA), AKT (1:1000; #4691S, Cell signaling technologies, Danvers, MA, USA), p-AKT (1:1000; #2965S, Cell signaling technologies, Danvers, MA, USA), p70s6 (1:1000; # 9202S, Cell signaling technologies, Danvers, MA, USA), p-p70s6 (1:1000; CS9205, Santa Cruz Biotechnology, Santa Cruz, CA, USA), Cyclin A (1:500, sc-239, Santa Cruz Biotechnology, Santa Cruz, CA, USA), Cyclin B1 (1:500, sc-245, Santa Cruz Biotechnology, Santa Cruz, CA, USA), PARP (1:1000, sc-7150, Santa Cruz Biotechnology, Santa Cruz, CA, USA), cleaved caspase-3 (1:500, #9505S, Cell signaling technologies, Danvers, MA, USA), cleaved caspase-9 (1:500, #9661S, Cell signaling technologies, Danvers, MA, USA), and BAX (1:1000, #5023S, Cell signaling technologies, Danvers, MA, USA) in 5% non-fat milk in TBS-T at 4 °C overnight. Next, matching secondary HRP-conjugated anti-rabbit or anti-mouse antibodies (1:3000 in 5% non-fat milk in TBS-T, 1 h, RT; Sigma, St. Louis, MO, USA) were employed. Immunoreactive bands were detected by an ECL™ kit (Pierce, Waltham, MA, USA) and exposed to radiographic film (AGFA, Mortsel, Belgium). Relative protein levels were quantified by a densitometry analysis using ImageJ (NIH, Bethesda, MD, USA).

### 2.7. Measurement of Metabolic Activity by BIOLOG MitoPlates

MitoPlates are 96-well microplates precoated with different mitochondria-specific substrates or inhibitors that are dried on the bottom of each well. Cells were incubated in a 25 mL flask at a concentration of 5 × 10^5^ cells/mL with pseurotin D (at concentrations of 15 and 25 µM) for 24 h. Saponin (final concentration 50 µg/mL) was used for the permeabilization of cells, and in the case of assays testing inhibitors, malic acid was used at a final concentration of 4 mM. According to the protocol, the assay mixture and cells (at a final concentration of 0.7 × 10^5^) were added to the wells. The color formation was read kinetically on an INFINITE microplate reader (Tecan, Mannedorf, Switzerland) at a wavelength of 590 nm.

### 2.8. Measurement of Mitochondrial Potential

Cells were incubated in a 24-well plate at a concentration of 2.5 × 10^5^ cells per well with pseurotin D (at concentrations of 10, 15, and 25 µM) for 6 and 24 h. The measurement of mitochondrial transmembrane potential was performed by red fluorescence dye TMRE (tetramethylrhodamine ethyl ester) staining [31]. TMRE accumulates in the functional mitochondrion, while a damaged mitochondrion loses potential and TMRE is washed out of the cells. A final concentration of 150 nM was used. Emitted fluorescence was detected by a flow cytometer (FACSVerse™, BD Bioscience, Carlsbad, CA, USA).

### 2.9. Measurement of Mitochondrial ROS Production

ROS production is one of the signs indicating disruption of the respiratory chain in mitochondria. To analyze mitochondrial ROS production, we used MitoSOX^TM^ to detect ROS production by mitochondria in whole cells, and Dihydrorhodamine 123 (DHR 123) and AmplexRed to detect ROS production in isolated mitochondria.

Cells were incubated in a 24-well plate at a concentration of 2.5 × 10^5^ cells per well with pseurotin D (at concentrations of 10, 15, and 25 µM) for 6 and 24 h. After incubation, a 5 µM MitoSOX^TM^ working solution was used. MitoSOX Red Mitochondrial Superoxide Indicator (Invitrogen, Waltham, MA, USA) is a fluorogenic dye that selectively targets mitochondria and is rapidly oxidized–specifically, by superoxide. The oxidized product is highly fluorescent upon binding to nucleic acid [32]. Emitted fluorescence was detected by a flow cytometer (FACSVerse™, BD Bioscience, Carlsbad, CA, USA).

Mitochondria were isolated according to the protocol by Frezza et al. [33]. Briefly, untreated cells were homogenized with a Teflon pestle in a glass potter and centrifuged (600 *g*/10 min/4 °C), and the supernatant containing isolated mitochondria was collected and centrifuged again (7000 *g*/10 min/4 °C). The pellet (isolated mitochondria) was used for further experiments. These isolated mitochondria were treated with pseurotin D (at concentrations of 10, 15, and 25 µM) and the production of ROS was detected by DHR 123 and AmplexRed within the next 45 min. Malic acid (2.5 mM) and succinate (5 mM) were used for the activation of mitochondria. Fluorescence was measured using an INFINITE microplate reader (Tecan, Mannedorf, Switzerland).

### 2.10. Measurement of Metabolic Activity by Seahorse Assay

Mitochondrial respiration, as a major energy-producing pathway for cells, was measured by an Agilent Seahorse XFp extracellular flux analyzer, as described previously in [24]. The Seahorse XF Cell Mito Stress Test Kit (Agilent, Santa Clara, CA, USA) reports multiple key parameters, including basal respiration, ATP-linked respiration, and spare respiratory capacity. To analyze metabolic activity by means of the Seahorse platform, cells were incubated in a 24-well plate at a concentration of 1.5 × 10^5^ cells per well; pseurotin D (1–25 µM) was added and incubated for 24 h at 37 °C under a 5% CO_2_ atmosphere. After the incubation period, cells were transferred into a poly-l-lysine coated 8-well plate (Agilent Seahorse XFp miniplate). Attached cells were washed with the assay medium, and a final volume of the assay medium was added and measured. The total amount of cells was used for data normalization. The Agilent Wave software was used for data evaluation. From these characteristic kinetic curves, basal respiration, proton leak, maximal respiration, and non-mitochondrial respiration were determined.

### 2.11. Statistical Analysis

Data are presented as mean ± standard error of the mean (SEM). The number of independent experiments (*n*) is stated in the figure legend. The data from some of the measurements were normalized to the control cells in each experiment to account for the variability of individual cell passages. Statistical analysis was performed using GraphPad Prism version 6.01 for Windows, GraphPad Software, La Jolla, CA, USA). Statistical differences were tested by one-way ANOVA combined with Dunnett’s multiple comparison test or by one sample *t*-test to compare values expressed as percentages. In the case of one sample *t*-test application, the Bonferroni correction of the *p*-value for multiple-comparisons was performed.

## 3. Results

### 3.1. Pseurotin D Decreased Cell Viability but Did Not Induce Acute Cytotoxicity in MEC-1 Cells

**Figure 1:**
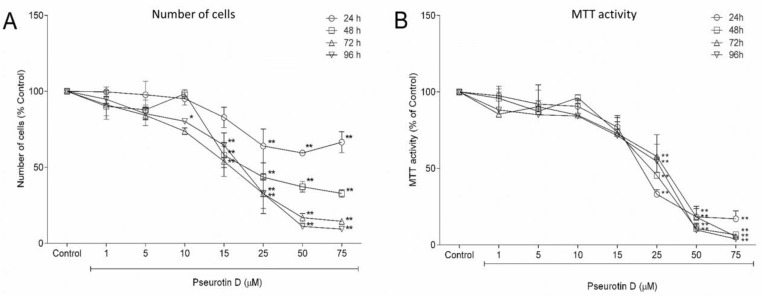
The effect of pseurotin D (1–75 µM) on MEC-1 proliferation and viability determined according to the number of cells (**A**) and MTT activity (**B**) after 24, 48, 72, and 96 h of incubation. Data are expressed as a percentage of the untreated control (mean ± SEM, *n* = 4). One sample *t*-test was used to analyze the significance of the obtained data separately comparing the effect of each compound with untreated control. Bonferroni correction of the *p*-value for multiple comparisons was performed (** *p* < 0.01 and * *p* < 0.05).

First, we investigated whether pseurotin D affected the viability of MEC-1 cells. For this purpose, MEC-1 cells were treated with a range of pseurotin D concentrations (1–75 μM) for 24, 48, 72, and 96 h. Pseurotin D significantly decreased the number of MEC-1 cells in a dose-dependent manner, with a calculated IC50 of 23 µM (Figure 1A). Specifically, pseurotin D at concentrations of 25, 50, and 75 μM significantly reduced the number of cells throughout the exposure period up to 9% of the control. Moreover, 10 and 15 μM concentrations of pseurotin D were able to significantly reduce the cell number after 48 h up to 73%, 58% of the control, respectively (Figure 1A). In addition to the direct counting of viable cells, the determination of metabolically active cells by MTT assay was used as another method for measuring viability. In accord with direct counting, pseurotin D (at concentrations of 25, 50, and 75 μM) significantly decreased the number of metabolically active cells after 24, 48, 72, and 96 h (Figure 1B). Moreover, pseurotin D had a greater effect on cell metabolic activity compared to control than the effect of pseurotin D on MEC-1 viability detected by cell counting (Figure 1A,B). Importantly, the decrease in MEC-1 cell viability and metabolic activity after pseurotin D exposure was not accompanied by any signs of acute toxicity, as shown by LDH release assay (Figure S2).

### 3.2. Pseurotin D Induced Apoptosis of MEC-1 Cells

**Figure 2:**
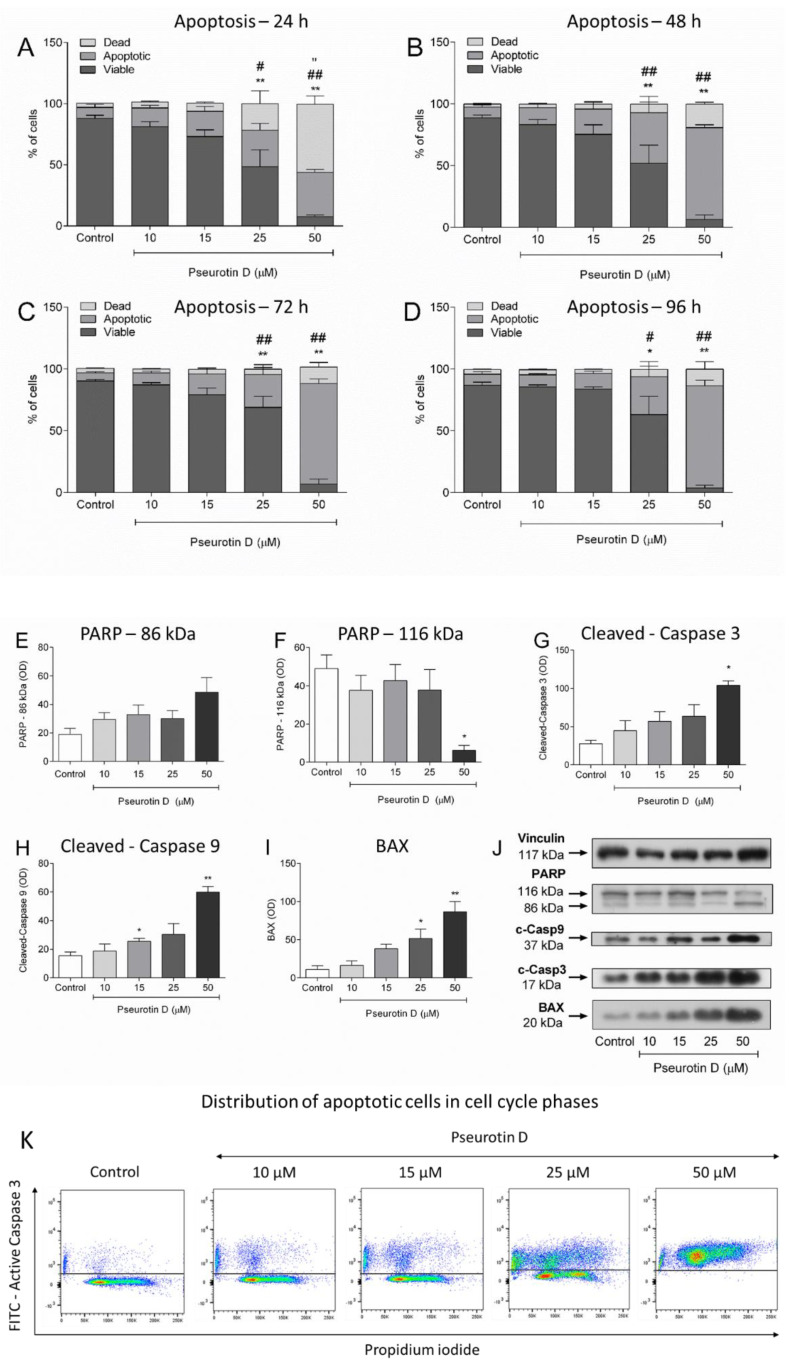
The effect of pseurotin D (1–50 µM) on the induction of MEC-1. The distribution of viable, apoptotic, and dead cells was determined on the basis of AnexinV/PI staining after 24, 48, 72, and 96 h (**A**–**D**). The level of cleaved caspase-3, cleaved caspase-9, PARP, BAX, and vinculin, as an equal loading control, were determined by Western blot analysis after 24 h (**E**–**J**). The distribution of apoptotic cells in cell cycle phases was determined by using antibody against active caspase-3 conjugated by FITC and PI staining (**K**). Data were expressed as the mean ± SEM (*n* = 3) and analyzed by ANOVA combined with Dunnett’s test (** *p* < 0.01 and * *p* < 0.05). In (**A**–**D**) * represents the statistical analysis of the viable cells, # represents the statistical analysis of the apoptotic cells, and “ represents the statistical analysis of the dead cells.

In light of the effects of pseurotin on cell viability, we focused on the capacity of pseurotin D in the concentration range 10–50 μM to induce apoptosis in lymphoma cells. Pseurotin D (25 and 50 μM) significantly increased the number of apoptotic cells and decreased the number of viable cells according to the surface expression of phosphatidylserine (determined by Annexin V staining) and cell membrane permeabilization (determined by PI staining) after 24, 48, 72, and 96 h (Figure 2A–D). Moreover, 50 μM of pseurotin D was able to significantly increase the number of dead cells up to 50% of control and decreased viable cells to 10% of control at all-time points of exposure (Figure 2A–D).

On the basis of these results, we focused on the 24-h exposure period and determined the levels of cleaved caspases 3 and 9, PARP and BAX. Similarly to the pseurotin D-mediated destabilization of cell membranes, pseurotin D increased the cleavage of caspase-3, -9, and PARP, which was statistically significant at 50 μM concentration (Figure 2E–J). Also, pseurotin D at a concentration of 25 μM significantly increased BAX protein (Figure 2I). Next, by using an antibody against the active form of caspase-3 and DNA content staining, we tested an approximate distribution of apoptotic cells in cell cycle phases (Figure 2K). Pseurotin at a concentration of 25 μM arrested the cells in the G2/M phase (Figures 2K and S3). However, an accumulation of active caspase-3 positive cells in any cell cycle phase was not evident since the distribution of DNA content in active caspase-3 positive cells was comparable to that of the live cells in control (Figure 2K). In addition, we looked at DNA fragmentation. The highest concentration of pseurotin D used, 50 μM, induced DNA fragmentation after 24 h and the lower concentration of pseurotin D, 25 μM, induced DNA fragmentation after 48 h (Figure S4).

### 3.3. Pseurotin D Arrested MEC-1 Cells in the G2/M Cell Cycle Phase

To gain a deeper insight into the pseurotin D-mediated inhibition of MEC-1 cell proliferation and possible arrest in G2/M cell phase, the cell cycle and expression of selected cyclins were evaluated after pseurotin D treatment. On the basis of previous results, we excluded the strong pro-apoptotic concentration of 50 μM. Therefore, we changed the concentration range of pseurotin D to 10–25 μM.

**Figure 3:**
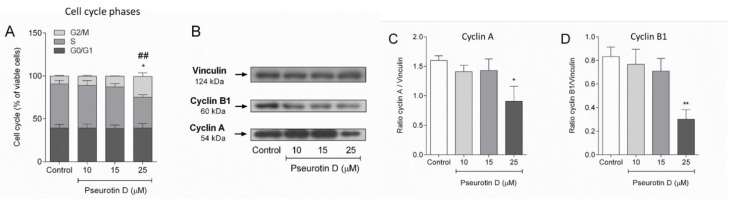
The effect of pseurotin D (10–50 µM) on the cell cycle. The cell cycle was analyzed by Clic-iT EdU kit (**A**). (**B**) shows a representative Western blot of the expression of cyclin B1, A, and vinculin by MEC-1 after 24 h of treatment. The analysis of optical density expressed as a ratio of the optical density of cyclins to the optical density of vinculin (equal loading control) is depicted in (**C**) for cyclin A and panel (**D**) for cyclin B1. Data were expressed as the mean ± SEM (*n* = 3) and analyzed by ANOVA combined with Dunnett’s test (** *p* < 0.01 and * *p* < 0.05). In (**A**) * represents the statistical analysis of the S phase and # represents the statistical analysis of the G2/M phase.

The results from the determination of DNA synthesis based on EdU incorporation showed that pseurotin D significantly decreased the number of cells in the S phase, increased the number of cells in the G2/M phase, but did not modify the number of cells in the G0/G1 phase (Figure 3A). Correspondingly, pseurotin D (25 µM) significantly decreased the expression of cyclin A, which regulates S/G2 phase transition, to approx. 60% of the protein expression in control cells (Figure 3B,C). The most substantial effect of pseurotin D was observed on cyclin B1, controlling G2/M phase transition, which was decreased to approx. 36% of the protein expression in control cells (Figure 3B,D).

### 3.4. Pseurotin D Decreased the Mitochondrial Respiration and Activity of MEC-1 Cells

Due to the significant effects of pseurotin D on cell metabolism determined by MTT, we studied the modulation of mitochondrial respiration, mitochondrial activity, and mitochondria transmembrane potential mediated by pseurotin D in MEC-1 cells.

**Figure 4:**
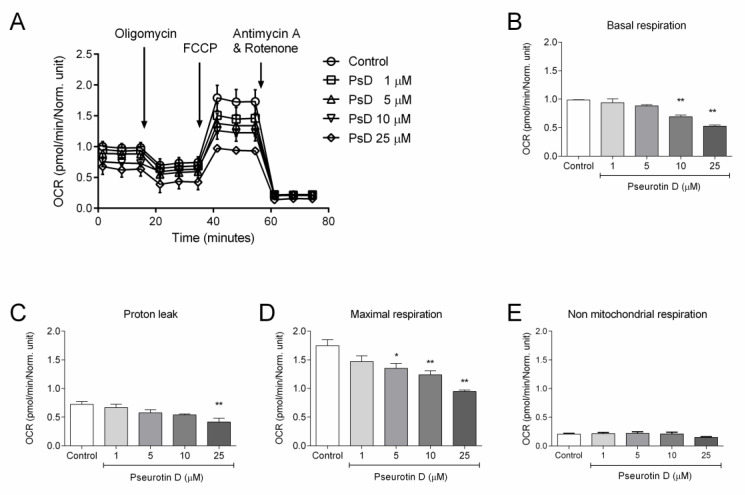
The effect of pseurotin D (1–25 µM) on MEC-1 metabolic activity. Cells were incubated with pseurotin D for 24 h and subsequently assessed with respect to their oxygen consumption rate (OCR). For induction of metabolic stress 1.0 µM oligomycin, 1.5 µM FCCP, 0.5 µM Antimycin A, and 0.5 µM Rotenone were used. Curves of OCR kinetics are shown in (**A**). Basal respiration (**B**), proton leak (**C**), maximal respiration (**D**), and non-mitochondrial respiration (**E**) were calculated from OCR kinetics using Wave Desktop 2.6 Software. OCR data were normalized to cell number by a CyQUANT probe and expressed as the mean ± SEM (*n* = 3) and analyzed by ANOVA combined with Dunnett’s test (** *p* < 0.01 and * *p* < 0.05).

Interestingly, overall mitochondria respiration corrected to the number of viable cells decreased after 24 h of exposure to pseurotin D (Figure 4A). Specifically, pseurotin D at the concentrations of 10 and 25 μM significantly reduced basal respiration from 1.00 to 0.69 and 0.52 pmol/minutes/norm. unit, respectively (Figure 4B). Pseurotin D at concentrations of 5, 10, and 25 μM significantly reduced maximal respiration from 1.75 to 1.35, 1.24, and 0.95 pmol/minutes/norm. unit, respectively (Figure 4D). Pseurotin D was able to significantly reduce proton leak only at the 25 μM concentration, this from 0.72 to 0.42 pmol/minutes/norm. unit (Figure 4C). In contrast, pseurotin D did not affect non-mitochondrial respiration at any of the tested concentrations (Figure 4E).

**Figure 5:**
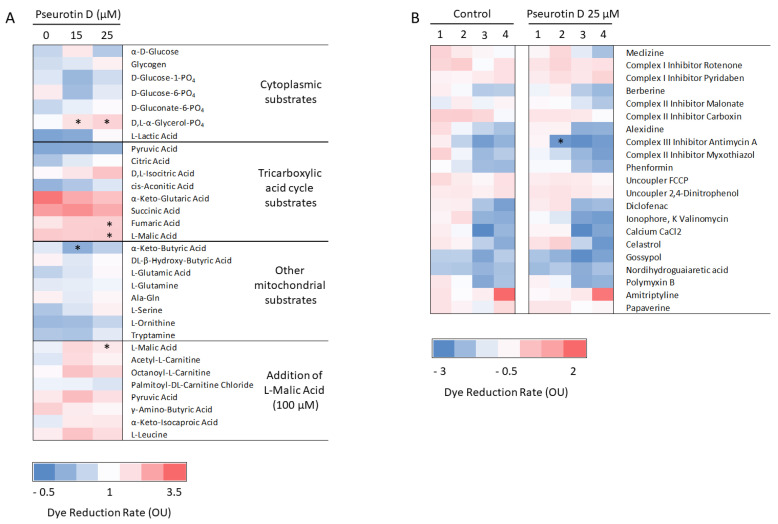
The effect of pseurotin D (15 and 25 µM) on MEC-1 mitochondrial metabolism. Cells were incubated with pseurotin D for 24 h. After the incubation period, the dye reduction rate over 3 h was measured using MitoPlate S-1 (**A**) and MitoPlate I-1 (**B**). MitoPlate S-1 contains 31 specific substrates or their combinations and MitoPlate I-1 contains 21 specific inhibitors in 4 different concentrations (marked with numbers 1–4). Data were expressed as the mean ± SEM (*n* = 3). One sample *t*-test was used to analyze the significance of obtained data separately comparing the effect of each compound with untreated control. Bonferroni correction of the *p*-value for multiple comparisons was performed (* *p* < 0.05).

In addition to the detection of mitochondria respiration, we tested mitochondrial function by using MitoPlates pre-coated with specific mitochondrial substrates or inhibitors. Pseurotin D was able to significantly upregulate formazan production in combination with substrates D, L-α-glycerol-PO4 (at both tested concentrations 15 and 25 μM), fumaric acid, L-malic acid (only at 25 μM), and α-keto-butyric acid (only at 15 μM). For other substrates, we observed changes compared to control, but they were not significant (Figure 5A). In addition, only 25 μM pseurotin D significantly reduced the rate of formazan reduction in combination with antimycin A (Figure 5B).

**Figure 6:**
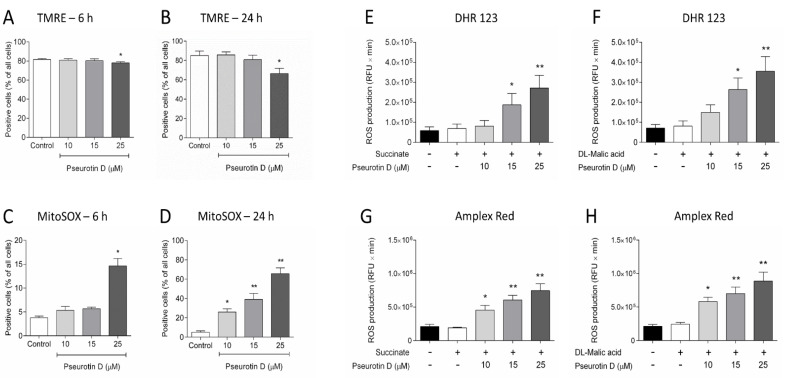
The effect of pseurotin D (10–25 µM) on MEC-1 mitochondrial membrane potential and mitochondrial ROS production. Cells were incubated with pseurotin D for 6 and 24 h, followed by an assessment of mitochondrial membrane potential (**A**,**B**) and mitochondrial ROS production (**C**,**D**). Isolated mitochondria were incubated with a combination of pseurotin D and substrate (succinate or malic acid) followed by the evaluation of ROS production using DHR-123 (**E**,**F**) and AmplexRed (**G**,**H**). Data were expressed as the mean ± SEM (*n* = 3) and analyzed by ANOVA combined with Dunnett’s test (** *p* < 0.01 and * *p* < 0.05).

In light of previous data, we focused directly on mitochondria redox balance, especially on the change in membrane potential and the production of ROS by mitochondria. Pseurotin D at a concentration of 25 μM was able to significantly decrease mitochondrial membrane potential after 6 and 24 h of incubation (Figure 6A,B). Moreover, at the same time points, 6 and 24 h, pseurotin D significantly increased mitochondrial ROS production (Figure 6C,D). After 6 h of incubation, the significantly effective concentration was only 25 μM and the increased ROS production was in approximately 15% of cells (Figure 6C). In contrast, after 24 h of incubation, all tested concentrations were able to significantly increase mitochondrial ROS production. Pseurotin D concentrations of 10 μM, 15 μM, and 25 μM increased ROS production in 26%, 39%, and 66% of the total number of cells, respectively (Figure 6D).

### 3.5. Pseurotin D Downregulated the Phosphorylation of Key Signaling Pathways in the Proliferation of Cells

**Figure 7:**
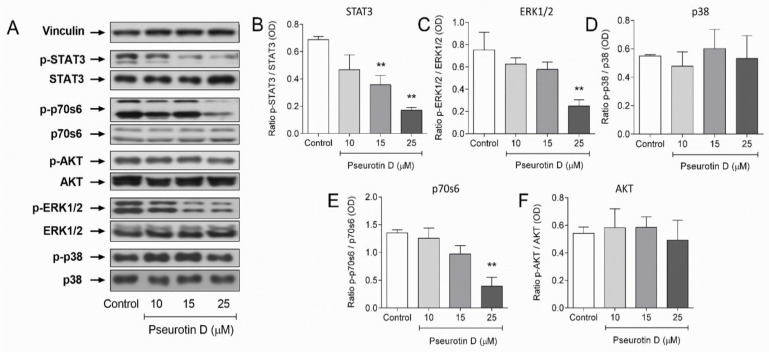
The effect of pseurotin D (10–25 µM) on the phosphorylation of selected signaling pathway proteins of MEC-1 cells. Protein expression and phosphorylation were analyzed by western blot and the analysis of optical density as a ratio of the optical density of the phospho form to the optical density of the total form were analyzed by GraphPad Prism software. (**A**) shows representative Western blots. Other panels show results from STAT3 (**B**), ERK1/2 (**C**), p38 (**D**), p70s6 (**E**), and AKT (**F**) phosphorylation. Data are expressed as the mean ± SEM (*n* = 3) and analyzed by ANOVA combined with Dunnett’s test (** *p* < 0,01 and * *p* < 0.05).

Finally, we focused on signaling pathways that are important in cell proliferation and tumorigenesis. One of the essential pathways of cancer cell proliferation is STAT signaling. Pseurotin D had a strong inhibitory effect on STAT3 phosphorylation. Even 15 and 25 μM pseurotin D significantly inhibited the phosphorylation of STAT3 protein to approx. 50 and 30% of untreated control after 6 h of incubation, respectively (Figure 7A,B). Other important signaling pathways are MAPK and AKT. After 6 h of incubation, pseurotin D significantly inhibited the phosphorylation of ERK1/2 to approx. 33% of untreated control (Figure 7A,C). The phosphorylation of p38 kinase was without significant change (Figure 7A,D). Interestingly, 25 µM pseurotin D was able to significantly inhibit p70s6 kinase but not AKT (Figure 7A,E,F).

## 4. Discussion

In this study, for the first time, we described the mechanism of action of pseurotin D on lymphoma cells, showing key effects on redox regulation associated with increased mitochondrial ROS production.

Pseurotin D downregulated the proliferation of MEC-1 cells without any acute toxic effects on these cells in concentrations up to 75 µM. Similarly, we previously showed that pseurotin and its analogs decreased the proliferation of T and B lymphocytes [17,25] and macrophages [24]. Further, Anjum et al. showed that pseurotin A has a significant suppressive effects on the proliferation of human glioma cell lines [23]. Specifically, we showed that pseurotin D arrested MEC-1 cells in the G2/M phase, this phenomenon accompanied by significantly decreased levels of cyclins A and B1. Further, in our study, we also demonstrated that pseurotin D induced the apoptosis of MEC-1 cells. In addition to these observations, we showed a significant increase in mitochondrial ROS production, which we consider to be the cause of cell cycle arrest and apoptosis. Similarly to this suggested mechanism, Chen et al. reported the ROS-mediated activation of apoptotic pathways, including the activation of caspase-9, caspase-8, and caspase-3 [34]. Related to this, the arrest of the cell cycle in the G2/M phase and apoptosis could be caused by the ROS-sensitive MAPK signaling pathway, as was shown in the saponin ginsenoside-mediated suppression of the growth of gastric cancer cells [35]. Likewise, Li et al. reported the induction of ROS-mediated G2/M phase arrest through inhibition of the NF-kappaB pathway in nasopharyngeal carcinoma [36].

In light of this inhibition of proliferation, we focused on the main signaling pathways affecting the proliferation of MEC-1 cells. Analysis of the JAK/STAT signaling pathway revealed the significant inhibition of STAT3 phosphorylation. Similarly, pseurotin D was able to inhibit the phosphorylation of ERK1/2 kinase, but not the p38 kinase. We observed similar results in our previous studies on T and B lymphocytes [17,25] and macrophages [24]. Moreover, Asami et al. reported that azaspirene, a compound from pseurotin family, was able to inhibit Raf-1, MEK1/2 and ERK1/2 phosphorylation after activation by VEGF in human endothelial cells [20]. The last signaling pathway we examined was the AKT signaling pathway. Our results showed that pseurotin D inhibited p70s6 kinase that acts downstream of the AKT signaling pathway and regulates protein synthesis [37]. On the other hand, we did not observe the inhibition of AKT phosphorylation. Therefore, the inhibition of p70s6 kinase could occur through direct inhibition by pseurotin or due to other kinases affecting mTOR regulating the activity of p70s6 kinase as well [37,38].

Interestingly, all mentioned pathways are also closely related to the regulation of mitochondrial functions. At the same time, these pathways are affected by changes in intracellular redox status driven most significantly by mitochondria [13,38,39,40,41,42]. Thus, we focused on mitochondria and mitochondria-mediated ROS production. After 24 h of incubation with pseurotin D, the basal and maximal respiration, but not the non-mitochondrial respiration of mitochondria, were significantly reduced. Moreover, after 24 h of incubation with pseurotin D, mitochondrial membrane potential was significantly reduced, and mitochondrial ROS production was significantly increased. Our results showed the significant inhibition of mitochondrial activity by pseurotin D in combination with selected substrates (e.g., fumaric and malic acids) which are important substrates of the tricarboxylic acid cycle. However, since the metabolism of other substrates related to tricarboxylic acid cycle were not affected by pseurotin D, the data did not suggest any significant inhibition of a specific metabolic pathway. Interestingly, Anjum et al. reported reduction in pyruvate kinase M2 and LDH5 expression, the key enzymes in the conversion of pyruvate to lactate after 48 h of 30 μM pseurotin A treatment in glioma U87-MG cell line [23]. Moreover, Anjum et al. showed that pseurotin A after 48 h increased levels of pyruvate dehydrogenase beta, adenosine triphosphate synthase beta, and cytochrome C, which are important enzymes in tricarboxylic acid cycle and oxidative phosphorylation [23]. However, these data should be considered with caution since they were recorded only after 48 h of incubation, and the metabolically active cells were not determined. In contrast with our observation, Chen et al. showed that pseurotin A can suppress intracellular RANKL-induced ROS production enhancing the expression of antioxidant enzymes, which attenuates the activation of MAPK [43]. This may be due to the fact that in this study, the authors used RANKL as an activator to increase intracellular ROS production and thus RANKL surpassed the effect of pseurotin itself on mitochondrial ROS production.

Overall, we showed for the first time that pseurotins are also useful in suppressing the proliferation of other non-solid cancers such as chronic lymphocytic leukemia (represented by the MEC-1 cell line) via mitochondria and the ROS-related caspase pathway. However, other mechanisms have also been suggested in two recent studies, these confirming the inhibitory effect of pseurotins also on solid tumors. Helal et al. showed the anti-carcinogenic effect of pseurotin A in hepatocellular carcinoma, this related to its anti-inflammatory, cytotoxic, and pro-apoptotic activities [44]. In addition, Abdelwahed et al. showed that pseurotin A suppressed hormone-dependent breast tumor progression and recurrence by targeting the PCSK9-LDLR axis [45].

## 5. Conclusions

It can be concluded that pseurotins are able to reduce the proliferation of MEC-1 cell line and also induce their apoptosis. These effects were related to pseurotin D mediated inhibition of STAT signaling, disturbance of mitochondria redox balance and activation of molecular mechanisms leading to apoptotic cell death. Our results highlight the fundamental role of natural small molecules which could represent a potent new group of compounds for the further development of suitable drugs for the treatment of lymphomas. However, further studies confirming our conclusions must be elucidated on lymphoma cells isolated from CLL patients and a suitable in vivo mouse model.

## Data Availability

The data are contained within the article and Supplementary Materials.

## Supplementary Materials

The following are available online at https://www.mdpi.com/article/10.3390/antiox10101576/s1. Figure S1: The effect of DMSO on MTT activity of MEC-1 cells; Figure S2: The potential effect of pseurotin D (1–75 µM) on MEC-1 cytotoxicity; Figure S3: The effect of pseurotin D on the distribution of apoptotic cells in cell cycle phases; Figure S4: The effect of pseurotin D (25 and 50 µM) on the fragmentation of nuclei.
