# Supplementary material for: Pseurotin D Induces Apoptosis through Targeting Redox Sensitive Pathways in Human Lymphoid Leukemia Cells
Source: Antioxidants (Basel). 2021 Oct 5;10(10):1576. doi: 10.3390/antiox10101576 (PMC8533295; doi:10.3390/antiox10101576)
Supplement: Supplementary file 1 [file antioxidants-10-01576-s001.zip › antioxidants-1371604-supplementary.pdf]

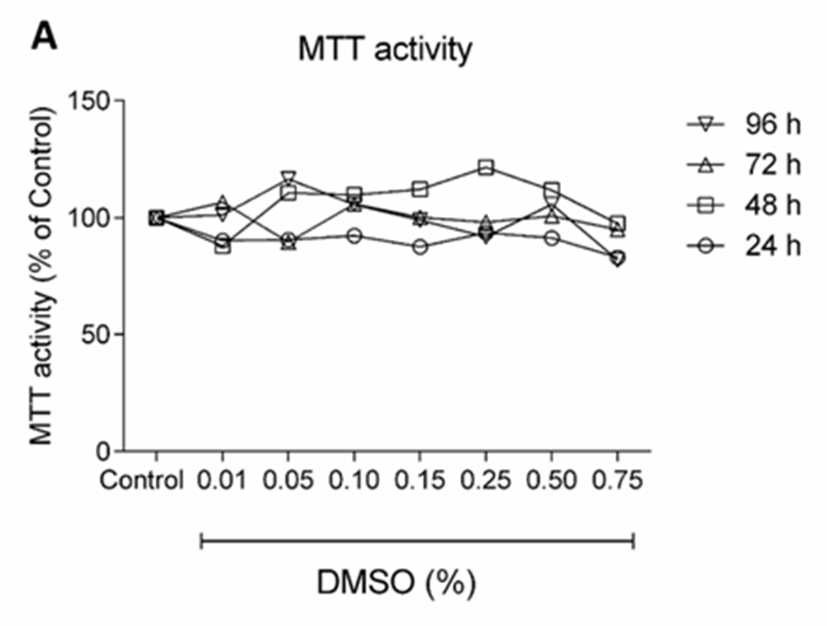


**Figure S1.** The effect of DMSO on MTT activity of MEC-1 cells after 24, 48, 72, and 96 hours of incubation. Data are expressed as a percentage of the untreated control.

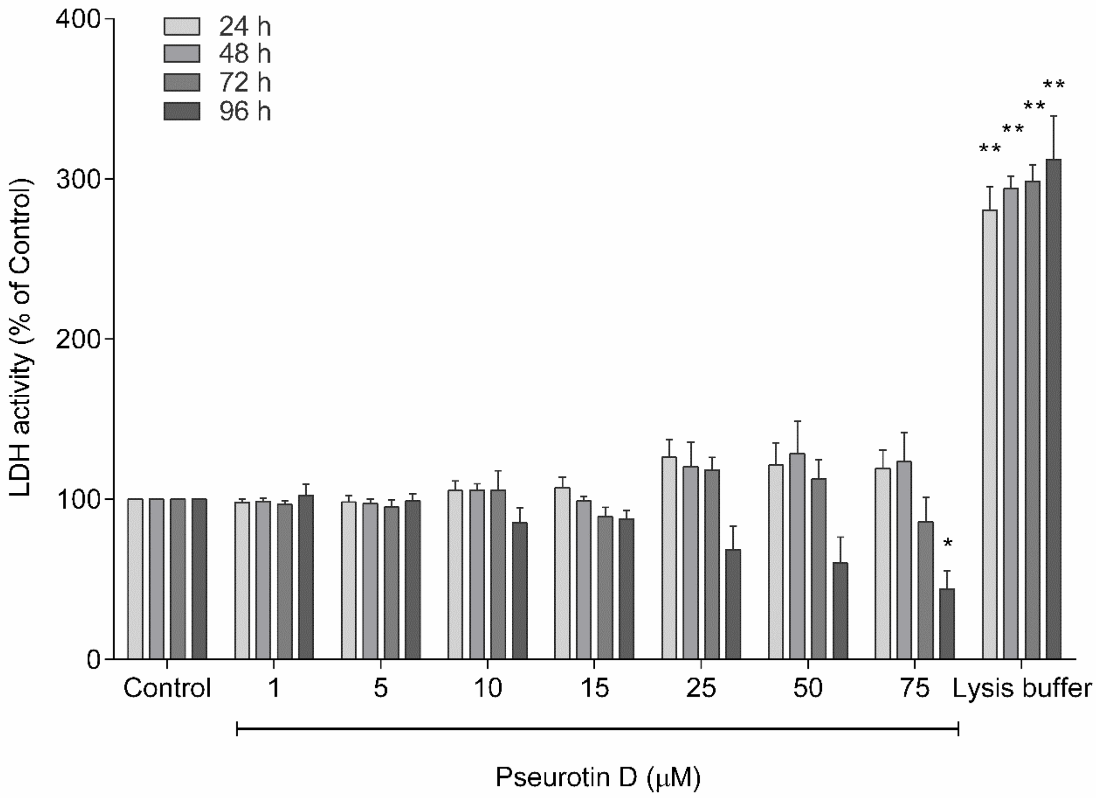


**Figure S2:** The potential effect of pseurotin D (1 – 75 μM) on MEC-1 cytotoxicity was determined on the basis of LDH release after 24, 48, 72, and 96 hours of incubation. In the case of LDH assay, detergent lysed cells were employed as positive control samples. Data were converted to a percentage of the control and expressed as the mean ± SEM (n = 4). One sample t-test was used to analyze the significance of obtained data separately comparing the effect of each compound with untreated control. Bonferroni correction of the p-value for multiple comparisons was performed (** $p < 0.01$ and * $p < 0.05$).

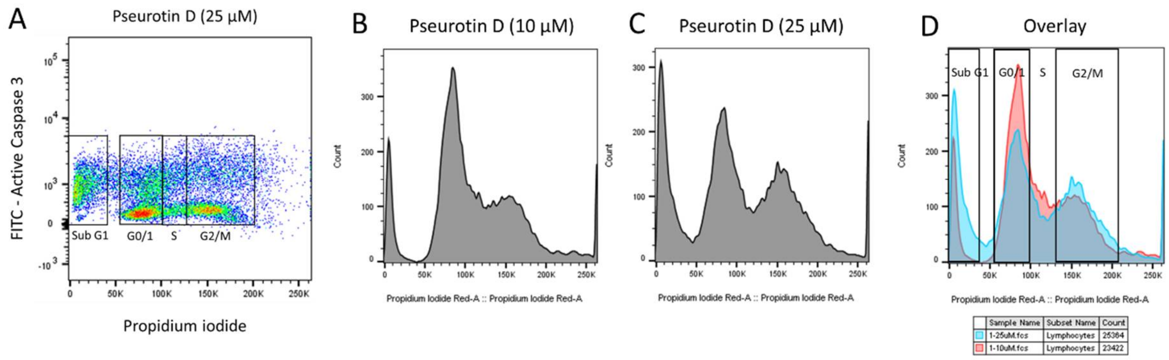


**Figure S3:** The effect of pseurotin D (10 and 25 µM) on the distribution of apoptotic cells (active caspase 3 positive cells) in cell cycle phases (panel A) was determined by using active caspase-3 conjugated by FITC and propidium iodide staining after 24 hours of incubation. Panel B and C show a histogram from PI staining and panel D shows an overlay of histograms and cell cycle distribution is shown.

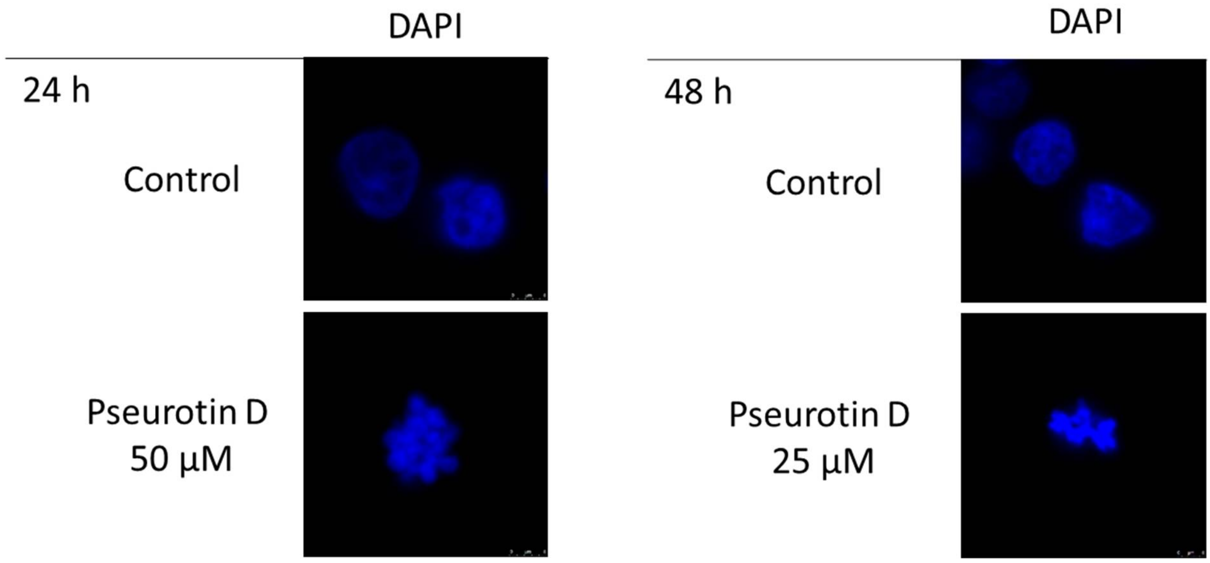


**Figure S4:** The effect of pseurotin D (25 and 50 µM) on the fragmentation of nuclei. The selected pictures of control cells and cells with fragmented nuclei after treatment by pseurotin D and 24 and 48 hours of incubation were obtained by confocal microscopy. The nuclei were stained with DAPI (blue color)..
